# Urothelium-derived prostanoids enhance contractility of urinary bladder smooth muscle and stimulate bladder afferent nerve activity in the mouse

**DOI:** 10.1152/ajpregu.00084.2024

**Published:** 2024-05-23

**Authors:** Thomas J. Heppner, Hannah J. Fallon, Jason L. Rengo, Elleanor M. Beaulieu, Grant W. Hennig, Mark T. Nelson, Gerald M. Herrera

**Affiliations:** ^1^Department of Pharmacology, University of Vermont, Burlington, Vermont, United States; ^2^Division of Cardiovascular Sciences, University of Manchester, Manchester, United Kingdom

**Keywords:** myogenic, overactive bladder, phasic contractility, prostaglandin

## Abstract

The transitional epithelial cells (urothelium) that line the lumen of the urinary bladder form a barrier between potentially harmful pathogens, toxins, and other bladder contents and the inner layers of the bladder wall. The urothelium, however, is not simply a passive barrier, as it can produce signaling factors, such as ATP, nitric oxide, prostaglandins, and other prostanoids, that can modulate bladder function. We investigated whether substances produced by the urothelium could directly modulate the contractility of the underlying urinary bladder smooth muscle. Force was measured in isolated strips of mouse urinary bladder with the urothelium intact or denuded. Bladder strips developed spontaneous tone and phasic contractions. In urothelium-intact strips, basal tone, as well as the frequency and amplitude of phasic contractions, were 25%, 32%, and 338% higher than in urothelium-denuded strips, respectively. Basal tone and phasic contractility in urothelium-intact bladder strips were abolished by the cyclooxygenase (COX) inhibitor indomethacin (10 µM) or the voltage-dependent Ca^2+^ channel blocker diltiazem (50 µM), whereas blocking neuronal sodium channels with tetrodotoxin (1 µM) had no effect. These results suggest that prostanoids produced in the urothelium enhance smooth muscle tone and phasic contractions by activating voltage-dependent Ca^2+^ channels in the underlying bladder smooth muscle. We went on to demonstrate that blocking COX inhibits the generation of transient pressure events in isolated pressurized bladders and greatly attenuates the afferent nerve activity during bladder filling, suggesting that urothelial prostanoids may also play a role in sensory nerve signaling.

**NEW & NOTEWORTHY** This paper provides evidence for the role of urothelial-derived prostanoids in maintaining tone in the urinary bladder during bladder filling, not only underscoring the role of the urothelium as more than a barrier but also contributing to active regulation of the urinary bladder. Furthermore, cyclooxygenase products greatly augment sensory nerve activity generated by bladder afferents during bladder filling and thus may play a role in perception of bladder fullness.

## INTRODUCTION

The wall of the urinary bladder is made up of a heterogeneous array of cell types, each of which plays a role in normal bladder function. Contraction and relaxation of urinary bladder smooth muscle (UBSM) underlie the important storage and voiding functions of the bladder. Under normal circumstances, UBSM is influenced by autonomic nerves that signal the bladder to contract and empty when the bladder is full (1). The lumen of the bladder is lined with a transitional epithelium, or urothelium, which undergoes drastic shape changes as the bladder fills and empties (2, 3). The urothelium is known to be a powerful barrier, preventing microorganisms, toxins, or environmental factors from disrupting the function of the organ (2, 4–6). In fact, the permeability of the urothelium is the lowest of any known epithelium (2).

It is generally accepted that the urothelium functions as more than a barrier to protect underlying tissue. The urothelium may play a sensory role in transmitting information about the filling status of the bladder and the presence of noxious compounds in the urine to nearby afferent nerves (7–12). There is some evidence suggesting that ATP released from urothelial cells in response to stretch as the bladder fills with urine activates purinergic receptors (P2X_3_) present on sensory neurons in the subepithelial nerve plexus. This process may contribute to the sensation that the bladder is full (9).

The urothelium may also release factors that directly modulate the contractility of urinary bladder smooth muscle. Nitric oxide (NO), ATP, and prostaglandins (PGs) have all been proposed to be released by the urothelium (10, 13, 14). Such factors may influence the contractility of urinary bladder smooth muscle directly, independent of neuronal activity, much like the vascular endothelium exerts a tonic relaxing effect on the vascular smooth muscle. However, previous studies have yielded conflicting results regarding modulation of urinary bladder smooth muscle contractility by the urothelium (11, 14–20).

PGs induce contractions of UBSM through several different PG receptors (21, 22). The PG-induced contractions appear to have tonic and phasic components, and the phasic contractions strongly resemble spontaneous transient contractions that are often observed in urinary bladder strips in vitro (21, 22). The constitutively expressed isoform of cyclooxygenase (COX), COX-1, is expressed in the bladder urothelium (23). Thus, it is possible that endogenous PGs could either directly cause or modulate spontaneous transient contractions in bladder strips. The spontaneous transient contractions of the detrusor have an important physiological function, as these contractions lead to transient pressure events in the whole bladder, and these transient pressure events give rise to bursts of afferent nerve activity (24).

In the present study, we hypothesized that the urothelium could exert a tonic modulatory effect on urinary bladder smooth muscle contractility, independent of efferent nerve activity. Since PGs are produced abundantly by the urothelium (25–28), we investigated the possibility that PGs or other COX products produced locally could modulate urinary bladder contractility. Our results indicate that urothelium-derived prostanoids enhance bladder smooth muscle contractility by promoting Ca^2+^ influx through voltage-dependent Ca^2+^ channels in UBSM and may also directly activate bladder sensory nerves to enhance sensory output during bladder filling.

## METHODS

### Animal Usage

All experimental protocols were reviewed and approved by the Institutional Animal Care and Use Committee of the University of Vermont. Male C57Bl6 mice (21.8–44.8 g body wt) were used for all experiments. Mice were euthanized by intraperitoneal injection of a lethal dose of euthanasia solution, followed by a thoracotomy. Urinary bladders were removed and placed in ice-cold dissection solution (see *Reagents and Solutions* for all solution compositions).

### Contractility Studies

The bladder was cut open to expose the urothelial surface and rinsed several times with dissection saline (see *Reagents and Solutions*) to remove residual traces of urine. The base of the bladder, including the neck and trigone region, was removed. Small strips of detrusor (2- to 3-mm wide and 5- to 7-mm long) were cut from the bladder wall. The urothelium was removed from alternating strips. Silk threads (6-0 silk suture; Fine Science Tools or Gossamer Silk Fly Tying Thread) were attached to each end of the strips, and the strips were transferred to cold (4°C) physiological saline solution (PSS).

**Figure 1.**
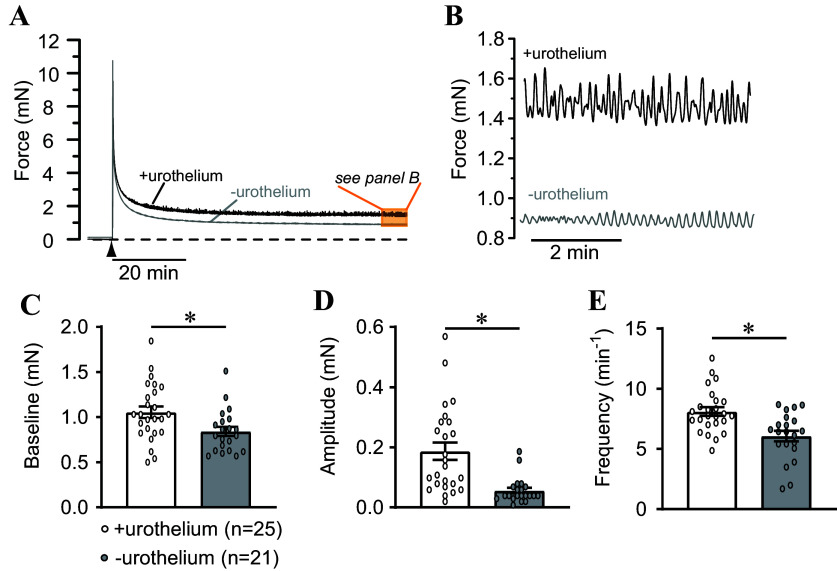
Development of stable tone and phasic contractions following stretch in urinary bladder strips. *A*: representative records showing stabilization of force following initial setting of load to ∼10–12 mN (▴) in urothelium-intact (+urothelium) and urothelium-denuded (–urothelium) bladder strips. Dashed line shows zero force. *B*: expanded region from orange highlighted box in *A* showing phasic contractions in the presence and absence of urothelium. *C*: summary data illustrating elevated basal tone present in urothelium-intact bladder strips. Phasic contraction amplitude (*D*) and frequency (*E*) were elevated in urothelium-intact strips relative to urothelium-denuded strips. Data are means ± SE with individual replicates indicated. *P < 0.05 unpaired *t* test.

Contractility of isolated bladder smooth muscle strips was measured using a MyoMED myograph system (MED Associates, Inc., Georgia, VT) or a custom laboratory-fabricated apparatus using Radnoti force transducers. Each strip was mounted in a tissue bath containing PSS (5–10 mL volume) aerated with 95% O_2_-5% CO_2_ maintained at 37°C. Strips were left to equilibrate for 50 min with no applied load. At this time, some strips were pretreated with indomethacin (10 µM) or tetrodotoxin (TTX; 1 µM). After 10 min, strips were stretched to 10–15 mN of tension. After this initial stretch, the muscle strips relaxed rapidly due to the elastic nature of the tissue. Thus, a period of 45–60 min was allowed for a stable level of force to be achieved (see Fig. 1*A*). After this stabilization period, in some strips, indomethacin (10 µM) was applied. After stabilization of the indomethacin-induced relaxation, the voltage-dependent Ca^2+^ channel blocker diltiazem (50 µM) was administered. The proportion of indomethacin-sensitive tone was calculated by expressing the indomethacin-sensitive relaxation as a percentage of the total relaxation observed following application of diltiazem in the presence of indomethacin. In another group of experiments, diltiazem (50 µM) was applied immediately after the initial 45- to 60-min stabilization period.

To characterize the effects of the urothelium on spontaneous phasic contractility of bladder strips, the amplitude and frequency of phasic contractions were measured over 5-min periods in each condition. Mini Analysis v6 Freeware (Bluecell, Seoul, Republic of Korea; http://bluecell.co.kr/theme/theme05/product/product_02_05.php) was used for analyzing phasic contractions.

Nerve-mediated contractions were studied using electric field stimulation (EFS) by a pair of electrodes parallel to the muscle strip mounted in the tissue bath. Urothelium-denuded strips were used for these experiments. Frequency-response curves were constructed by measuring the EFS-induced contraction amplitude as the stimulus pulse frequency was increased from 1 to 50 Hz. Pulse amplitude was 20 V, and polarity was reversed from pulse-to-pulse to prevent electrode polarization. Pulse width was 0.2 ms, and stimulus duration was 2 s. Stimuli were given every 3 min.

### Pressurized Bladder Preparation

Mice were euthanized as described in *Animal Usage*. The urinary bladder with urethra and ureters were carefully removed and placed in cold dissection solution. Both ureters were tied with 5/0 silk suture close to the bladder wall. The preparation was then placed in a recording chamber and superfused with PSS maintained at 37°C. pH was maintained at 7.4 by bubbling the solution with 20% O_2_-5% CO_2_-75% N_2_. The bladder was cannulated via the urethra and attached to a syringe pump (model 4400-001; Harvard Apparatus) allowing for continuous infusion of PSS into the bladder and an in-line pressure transducer (PT-F; Living Systems Instrumentation, Saint Albans, VT) connected to a signal conditioner (model NL-108; Digitimer, Hertfordshire, UK) set to 100 mV/cmH_2_O output for recording bladder pressure. Data acquisition was performed using a Power3A analog-to-digital converter and Spike2 software (Cambridge Electronic Design, Cambridge, UK) at a rate of 100 samples per second. Urinary bladders were filled at 1.8 mL/h to a pressure of 25 mmHg. Once this pressure was reached, the bladder was emptied through the urethral cannula. The maximum bladder pressure chosen was based on cystometry recordings from normal C57Bl/6 mice, which indicated that voiding threshold was reached at ∼11 mmHg and peak contraction pressures occurred at ∼26 mmHg (29). At least three successive filling/emptying cycles were performed to obtain stable urodynamic profiles. A stabilization period of 5 min is allowed between filling cycles. In this model, we define bladder capacity as the volume at which the bladder reaches a pressure of 25 mmHg. Bladder capacity increases slightly over time and usually stabilizes after the third filling cycle. Thus, we take the bladder capacity reached during the third cycle as 100%. We then fill the bladder to a volume of 80% and allow the pressure to stabilize for ∼30 min. During this time, transient pressure events (24) usually develop and stabilize in frequency and amplitude. Indomethacin (10 µM) was then applied to the tissue bath. After 30- to 45-min incubation in indomethacin, the calcium channel blocker diltiazem (50 µM) was applied to the bathing solution. Transient pressure events were quantified using peak detection features in Spike2 software (apply smoothing to pressure signal with a time constant of 0.5 s followed by Spike2 “Peak Find” function with an amplitude threshold of 0.08 mmHg).

### Ex Vivo Pressurized Bladder with Afferent Nerve Recordings

Bladders were prepared as described in *Pressurized Bladder Preparation*, except that the postganglionic nerves, major pelvic ganglia, and pelvic nerves were also removed. The pelvic nerves were carefully cleaned of connective tissue after tying off both ureters. The preparation was then placed in a recording chamber and superfused with PSS (37°C, pH 7.4 by bubbling with 20% O_2_-5% CO_2_-75% N_2_). The bladder was then cannulated via the urethra and attached to a variable-speed syringe pump (KD Scientific; model KDS-100) to allow for continuous infusion of PSS into the bladder and in-line pressure transducer (model PS-200; Living Systems Instrumentation, St. Albans, VT) for measuring bladder pressure. One of the pelvic nerves was then placed in a glass fire-polished tip (tip opening, ∼100 μm) of a suction electrode using negative pressure for recording afferent nerve activity from the urinary bladder.

Action potentials recorded from the pelvic nerve were collected using a Neurolog Headstage (NL100AKS; Digitimer, Hertfordshire, UK), amplified with an AC preamplifier (NL104; Digitimer), and band-pass filtered at 200–4,000 Hz (NL125/NL126; Digitimer) to remove noise. Data were collected and stored using a Power 401 analog-to-digital interface and Spike 2 software (Cambridge Electronic Design, Cambridge, UK). Pressure was acquired at a rate of 100 Hz, and afferent nerve activity was acquired at a rate of 25,000 Hz. The threshold for detection of bladder afferent nerve activity was set to ≤10 Hz when the bladder was empty. Bladder pressure and afferent nerve activity were recorded simultaneously and analyzed offline using Spike 2 software (Cambridge Electronic Design) and custom software written using MATLAB (MathWorks, Natick, MA). Action potential frequency was calculated from detected action potentials. “Afferent activity” was defined as the number of action potentials per second (Hz). The baseline pressure and afferent nerve activity signals were measured by fitting pressure and nerve time-series data using an asymmetric least squares smoothing function published by Eilers and Boelens (30) with a smoothing term of 10^−12^ and an asymmetry term of 0.01.

As before, at least three successive filling cycles were performed by infusing PSS into the bladder at a rate of 1.8 mL/h until bladder pressure reached 25 mmHg. At this point, indomethacin (10 µM) or its vehicle (ethanol, 0.01%) was applied into the lumen of the bladder by including it in the PSS in the syringe pump as well as into the bath. Filling/emptying cycles continued to be performed for 30–45 min in the presence of indomethacin or its vehicle.

### Reagents and Solutions

Euthanasia solution (Euthasol) was obtained from Midwest Veterinary Supply and contained pentobarbital sodium (390 mg/mL), phenytoin sodium (50 mg/mL), 10% ethanol, 18% propylene glycol, rhodamine B (0.003688 mg/mL), and 2% benzyl alcohol (preservative) in water. The pH is adjusted (by the manufacturer) using sodium hydroxide or hydrochloric acid. All other drugs and reagents were from Sigma. Dissection solution consisted of (in mM) 80 monosodium glutamate, 55 NaCl, 6 KCl, 10 glucose, 10 *N*-2-hydroxyethylpiperazine-*N'*-2-ethanesulfonic acid, 2 MgCl_2_, and pH adjusted to 7.3 with NaOH. PSS contained (in mM) 119 NaCl, 4.7 KCl, 24 NaHCO_3_, 1.2 KH_2_PO_4_, 2.5 CaCl_2_, 1.2 MgSO_4_, 11 glucose, and aerated with 95% O_2_–5% CO_2_ to obtain pH 7.4. HEPES-PSS solution consisted of (in mM) 134 NaCl, 6 KCl, 1 MgCl_2_, 2 CaCl_2_, 10 HEPES, and 7 glucose (pH 7.4 with NaOH).

### Data Analysis

GraphPad Prism (GraphPad Software, Boston, MA) was used for all statistical analyses. *P* < 0.05 was used as the threshold for null hypothesis rejection. An unpaired *t* test was used to compare means between urothelium-intact and urothelium-denuded strips. Comparisons between urothelium-intact and denuded strips in the presence of indomethacin and diltiazem were made using a repeated-measures two-way analysis of variance (ANOVA) followed by Šidák’s test for multiple comparisons. Transient pressure events in pressurized bladder preparations before and after indomethacin treatment were compared using a paired *t* test. Afferent nerve frequency before and after indomethacin (or vehicle) treatment in ex vivo pressurized bladder preparations was analyzed using two-way repeated measures analysis of variance followed by Šidák’s test for multiple comparisons. Summary data are presented as means ± SE. The number of experimental replicates (*n*) is stated in each figure and corresponds to the number of independent bladder strips or pressurized bladders examined from “*n*” number of mice.

## RESULTS

### Enhanced Contractility in Urothelium-Intact Bladder Strips

Isometric contractility was measured in urothelium-intact and denuded bladder strips. All bladder strips developed tone and phasic contractions (Fig. 1, *A* and *B*). To assess the baseline level of tone in urothelium-intact and denuded strips, force was averaged over a 2-min period of 60 min following the initial stretch (Fig. 1). Baseline force was 25% higher in urothelium-intact strips than in urothelium-denuded strips (Fig. 1*C*). Phasic contraction amplitude was also elevated in strips with intact urothelium (338% compared with urothelium-denuded strips, Fig. 1*D*), and phasic contraction frequency was increased by 32% in urothelium-intact strips compared with urothelium-denuded strips (Fig. 1*E*).

During dissection, strips were cut to approximately the same dimensions, but the urothelium-denuded strips may have incurred additional damage during the dissection procedure. To test whether both types of strips retained similar functionality, maximal contraction strength in response to EFS was assessed in urothelium-intact and denuded strips. EFS-induced contractions plateau at stimulation frequencies above 30 Hz (see Fig. 3); therefore, EFS-induced contractions were recorded using a 50-Hz stimulus for 2 s to assess maximal EFS-induced contractility. The maximal EFS-induced contraction amplitude was 23.5 ± 1.6 mN in urothelium-intact bladder strips (*n* = 5) and 28.5 ± 3.9 mN in urothelium-denuded strips (*n* = 5), an insignificant difference (*P* > 0.05). Contractility of urothelium-intact and denuded strips was also determined in response to elevated external KCl (60 mM KCl). KCl-induced contraction amplitudes were 3.7 ± 0.7 mN (*n* = 5) and 2.8 ± 0.6 mN (*n* = 6) in urothelium-intact and denuded strips, respectively, and not significantly different from each other (*P* > 0.05). Thus, urothelium-intact and denuded bladder strips responded in a similar manner to electrical and ionic challenges ruling out any significant effect that the dissection procedure might have had on their responsiveness.

**Figure 2.**
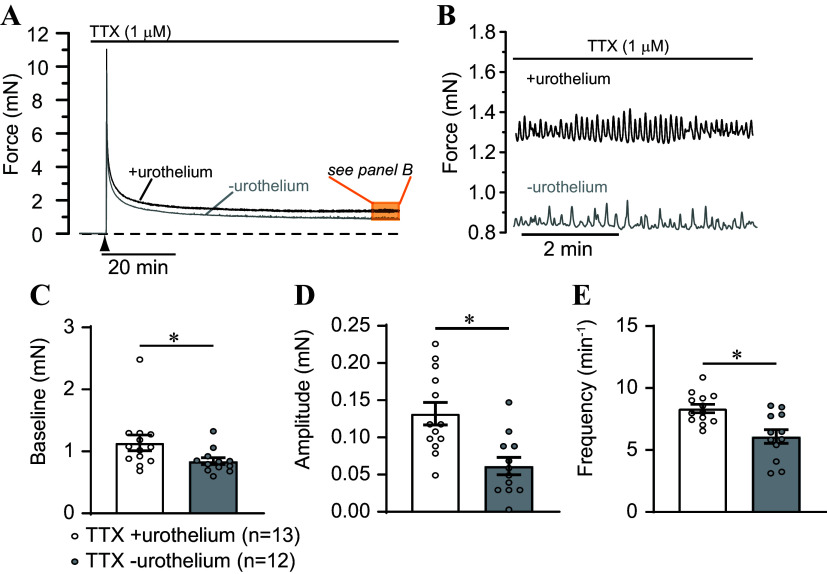
Non-neuronal basis for elevated contractility in urothelium-intact bladder strips. *A*: representative records showing force following initial setting of load to ∼10–12 mN (▴) in urothelium-intact (+urothelium) and urothelium-denuded (–urothelium) bladder strips in the presence of the neuronal Na^+^ channel blocker tetrodotoxin (TTX, 1 µM). Dashed line shows zero force. *B*: expanded region from orange highlighted box in *A* showing phasic contractions in TTX-treated strips in the presence and absence of urothelium. *C*: summary data illustrating elevated basal tone in urothelium-intact bladder strips. Phasic contraction amplitude (*D*) and frequency (*E*) were elevated in urothelium-intact strips relative to urothelium-denuded strips. Data are means ± SE with individual replicates indicated. *P < 0.05 unpaired *t* test.

**Figure 3.**
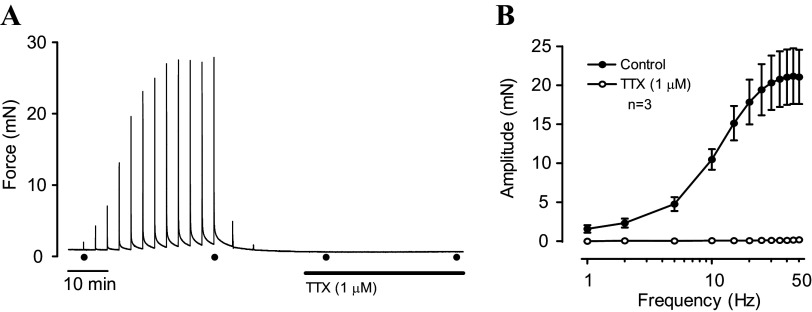
Inhibition of nerve-evoked UBSM contractions by TTX. *A*: original recording of EFS-induced contractions in an urothelium-denuded bladder strip before and after addition of TTX. The first and last stimuli in each series are indicated (●). *B*: EFS frequency–response curves obtained from experiments like those in *A*. TTX completely abolished EFS-mediated contractions. EFS, electric field stimulation; TTX, tetrodotoxin; UBSM, urinary bladder smooth muscle.

It is also possible that contractility may be affected by substances released from efferent nerves (31). To address this potential issue, urothelium-intact and denuded bladder strips were pretreated with the nerve toxin, tetrodotoxin (TTX, 1 µM; Fig. 2), a blocker of voltage-dependent sodium channels. TTX had no effect on tone or phasic contractions in either urothelium-intact or denuded strips (Fig. 2, *A* and *B*). Although baseline tension was 35% greater in TTX-treated urothelium-intact strips than in denuded strips (Fig. 2*C*), and phasic contraction amplitude and frequency were elevated by 214% and 37%, respectively (Fig. 2, *D* and *E*), these changes parallel the contractility responses in the absence of TTX where baseline tension was 25% higher, amplitude was 334% higher, and frequency was 32% higher in urothelium-intact strips compared with denuded strips, respectively (compare Fig. 1, *A* and *B* and Fig. 2, *A* and *B*). The efficacy of TTX was verified by its ability to inhibit nerve-mediated contraction (Fig. 3). TTX (1 µM) completely abolished EFS-induced contractions, indicating that it effectively eliminated nerve-mediated contractions in this preparation (Fig. 3). These findings indicate that the primary influence of the urothelium on bladder strip contractility was non-neuronal in nature.

### Urothelium-Derived Prostanoids Enhance Contractility of Urinary Bladder Smooth Muscle

**Figure 4.**
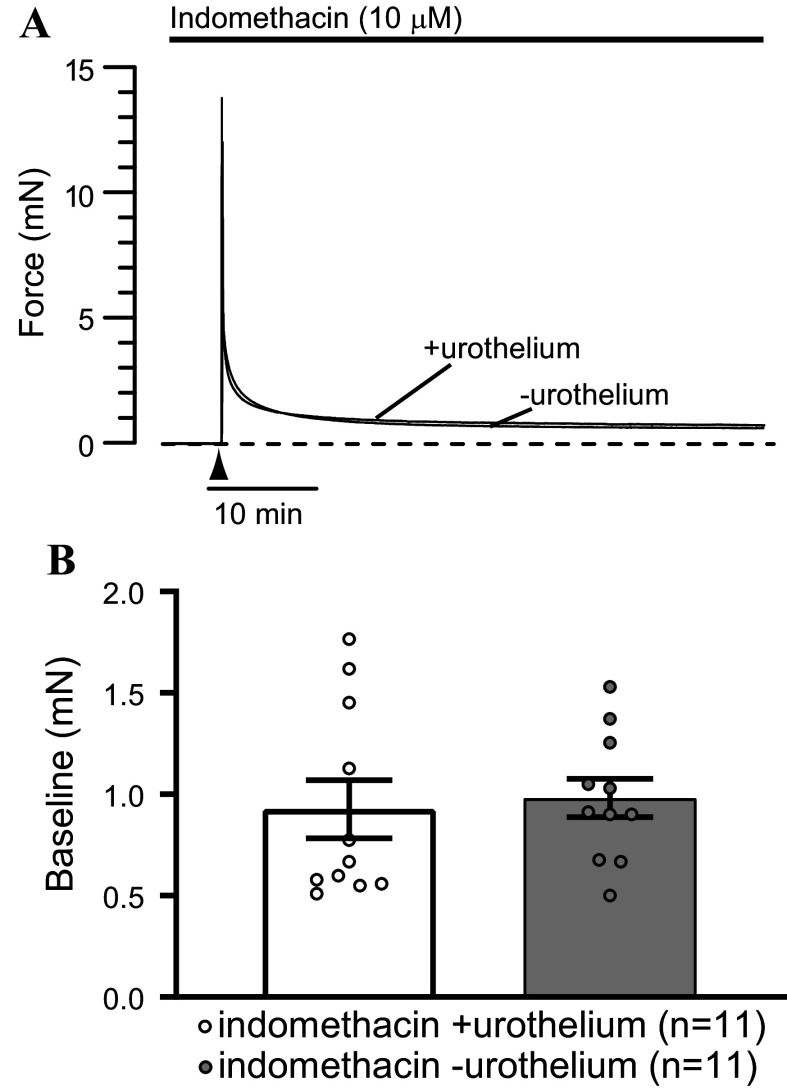
Urothelium-dependent increase in contractility is abolished by cyclooxygenase inhibition. *A*: representative recordings of force in indomethacin-pretreated bladder strips in the presence and absence of urothelium. Initial stretch to ∼14 mN was applied at the time indicated (▴). Dashed line shows zero force. *B*: there was no difference in the basal tone in indomethacin-treated urothelium-intact or denuded strips (*P* > 0.05, unpaired *t* test). Results are means ± SE with individual replicates indicated.

Bladder tissue has been reported to be a rich source of prostaglandins (32), and the urothelium is a major site of prostaglandin synthesis in the bladder wall (25, 27). Various prostaglandins, including F_2_α, D_2_, and E_2_, have been shown to cause bladder smooth muscle contractions (33). Therefore, we hypothesized that urothelium-derived prostanoids may underlie the urothelium-dependent increase in basal tone and phasic contraction amplitude and frequency in urinary bladder strips. To test this hypothesis, bladder strips were pretreated with the COX inhibitor indomethacin (10 µM) before stretching (Fig. 4). Pretreatment with indomethacin largely abolished phasic contractions (Fig. 4). Only 2 of 11 urothelium-intact and 2 of 11 urothelium-denuded bladder strips pretreated with indomethacin developed phasic contractions as opposed to 25 of 25 and 21 of 21 urothelium-intact and denuded strips not treated with indomethacin, respectively. Furthermore, there was no difference in the basal tone in urothelium-intact and urothelium-denuded bladder strips in the presence of indomethacin (Fig. 4*B*). These observations indicate that phasic contractions of UBSM rely on some level of prostaglandins or other COX products for their generation or expression.

Indomethacin-resistant tone was assessed by applying the voltage-dependent Ca^2+^ channel blocker diltiazem (50 µM) to indomethacin-pretreated strips. The magnitude of indomethacin-resistant tone was similar in urothelium-intact and denuded strips, as application of diltiazem caused a relaxation of 0.06 ± 0.01 and 0.10 ± 0.01 mN in urothelium-intact and denuded strips, respectively (*P* > 0.05). Thus, blocking prostaglandin synthesis with indomethacin eliminated the increase in basal tone and largely inhibited phasic contractions observed in urothelium-intact mouse urinary bladder strips.

**Figure 5.**
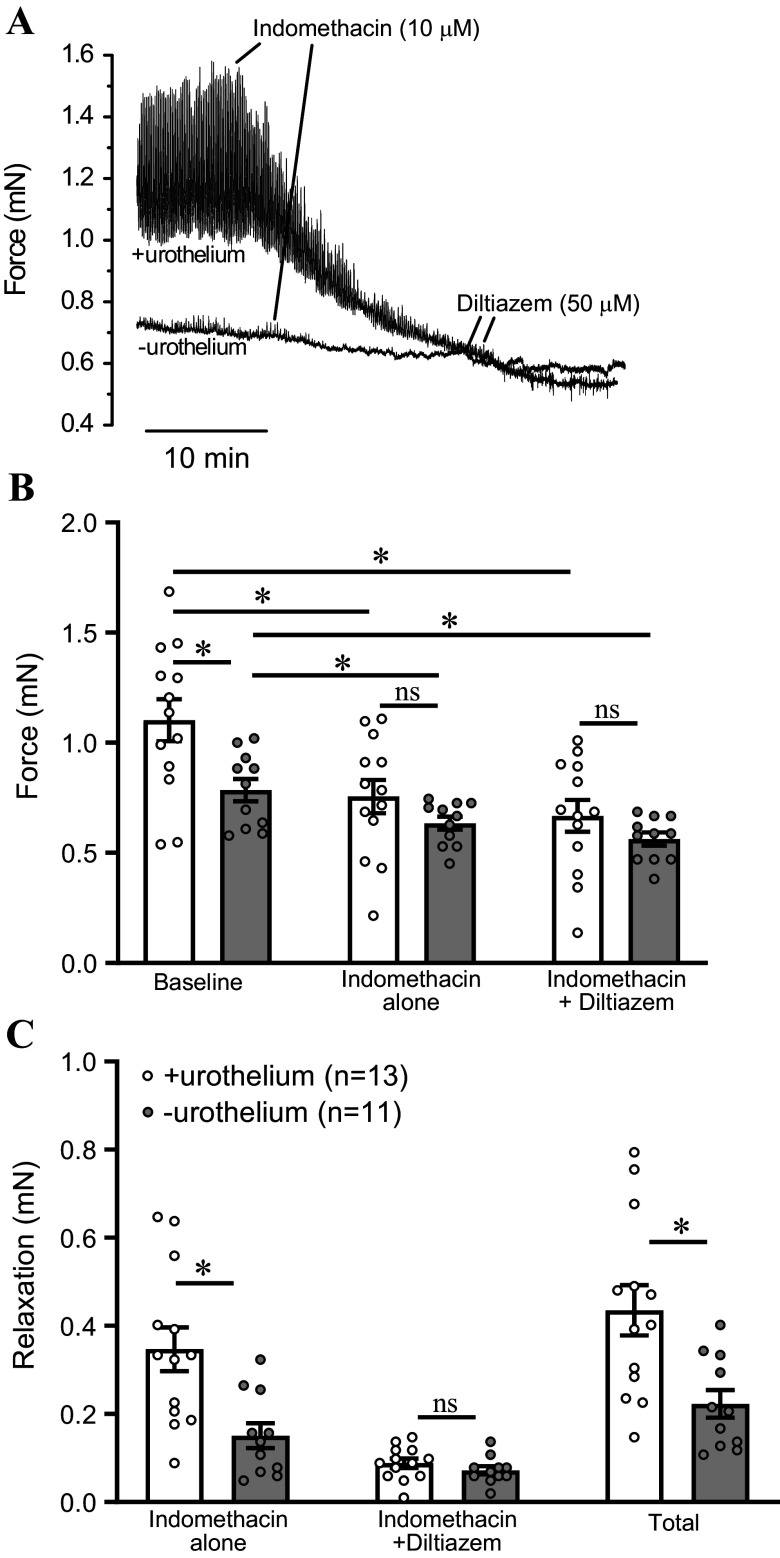
Indomethacin-sensitive tone is increased in urothelium-intact bladder strips. *A*: original data showing protocol for assessment of indomethacin-sensitive tone. Upon development of a stable relaxation response to indomethacin (10 µM), the voltage-dependent Ca^2+^ channel inhibitor diltiazem was applied (50 µM). The total relaxation observed in the presence of indomethacin and diltiazem was defined as 100% tone. *B*: average force during the three experimental conditions shown in *A*. Results are means ± SE with individual replicates indicated. *P < 0.05 for indicated comparison; two-way repeated measures ANOVA with Šidák’s test for multiple comparisons. *C*: relaxation responses calculated from data in *B*. Relaxation to indomethacin alone is found by subtracting the level of tone in the presence of indomethacin from the baseline tone. Relaxation to indomethacin and diltiazem is found by subtracting the level of tone in the presence of indomethacin and diltiazem from the level of tone in the presence of indomethacin. Total relaxation is found by adding the two preceding relaxation responses, or by subtracting the level of tone in presence of diltiazem and indomethacin from the baseline level of tone. Total relaxation (in the presence of indomethacin and diltiazem) was significantly greater in urothelium-intact strips than in denuded strips. The difference was due to the indomethacin-sensitive component of the relaxation being greatly increased in urothelium-intact strips. *P < 0.05 for indicated comparison; two-way repeated-measures ANOVA with Šidák’s test for multiple comparisons. ns, not significant.

The inhibitory effect of indomethacin was also examined in urothelium-intact and denuded bladder strips following development of tone and phasic contractions (Fig. 5*A*). Bladder strips that exhibited phasic contractions were treated with indomethacin (10 µM). As before, baseline force was increased in urothelium-intact strips relative to urothelium-denuded strips (Fig. 5*B*). Indomethacin induced a substantial relaxation response in urothelium-intact strips, and a smaller relaxation in urothelium-denuded strips (Fig. 5, *B* and *C*). In urothelium-intact strips, indomethacin decreased force by 0.35 ± 0.05 mN, whereas in urothelium-denuded strips indomethacin caused a relaxation of only 0.14 ± 0.03 mN (Fig. 5*C*, *P* < 0.05). Upon development of a stable relaxation in response to indomethacin, the voltage-dependent Ca^2+^ channel blocker diltiazem (50 µM) was applied to assess indomethacin-resistant tone (Fig. 5*A*). Inhibition of Ca^2+^ channels caused relaxation of urothelium-intact and denuded bladder strips (Fig. 5). In the presence of indomethacin, there was no difference between the diltiazem-induced relaxation in urothelium-intact or denuded strips (Fig. 5*C*). The total decrease in force following indomethacin and diltiazem treatment was defined as 100% tone. Thus, the indomethacin-sensitive component of the total tone was substantially elevated in urothelium-intact strips compared with urothelium-denuded strips (78.1 ± 2.7% vs. 61.2 ± 5.3%, *P* < 0.05). These findings suggest that urothelium-derived prostanoids enhance the development of stable tone by the detrusor.

### Role of Voltage-Dependent Ca^2+^ Channels in Enhanced Contractility of Urothelium-Intact Bladder Strips

**Figure 6.**
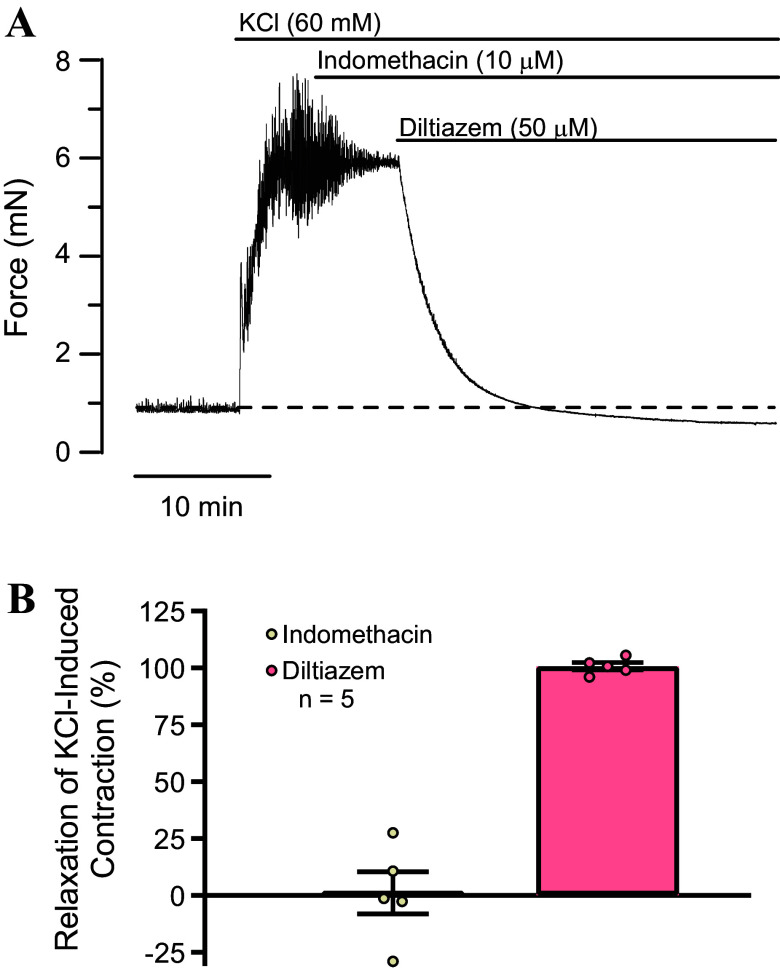
Indomethacin does not affect KCl-induced tonic contractions. *A*: recording of KCl-induced contraction in an urothelium-intact mouse bladder strip. KCl (60 mM) was administered to the bathing solution. After 5 min, indomethacin (10 µM) was applied. Phasic contractions were abolished by indomethacin, but the tonic contraction remained unaltered. Diltiazem (50 µM) completely reversed the KCl-induced contraction. Dashed line indicates basal tension before application of KCl. *B*: summary data showing percent reversal of KCl-induced contractions in urothelium-intact strips treated with indomethacin and diltiazem. Data are means ± SE with individual replicates indicated.

Phasic contractions of urinary bladder smooth muscle strips are dependent upon Ca^2+^ influx through voltage-dependent Ca^2+^ channels (34–36). One explanation for our present findings is that indomethacin inhibits phasic contractility not because it interferes with prostaglandin production, but because it blocks voltage-dependent Ca^2+^ channels (37). To address this possibility, bladder strips were contracted by elevating the concentration of KCl in the bathing medium to 60 mM (Fig. 6). Raising extracellular K^+^ concentration depolarizes smooth muscle, activates voltage-dependent Ca^2+^ channels, and causes contraction (38). Since K^+^-induced contractions require Ca^2+^-influx through voltage-dependent Ca^2+^ channels, if indomethacin were to block these channels, it should attenuate contractions. When 60 mM KCl was applied to urinary bladder strips, the response often consisted of phasic contractions superimposed on a tonic contraction (Fig. 6*A*). Indomethacin (10 µM) was applied at the steady-state of KCl-induced contractions (Fig. 6*A*). Phasic contractions were abolished by indomethacin, but tonic contractions remained (*n* = 5). Applying the voltage-dependent Ca^2+^ channel blocker diltiazem (50 µM) in the presence of indomethacin completely abolished the KCl-induced tone, and even revealed underlying basal tone (Fig. 6, *A* and *B*). Since indomethacin abolished the phasic contractions without affecting KCl-induced tone, it is unlikely that the elimination of phasic contractions by indomethacin is due to nonspecific inhibition of voltage-dependent Ca^2+^ channels by indomethacin.

To assess the link between prostaglandins and voltage-dependent Ca^2+^ channels, a separate series of experiments was performed in which urothelium-intact and denuded bladder strips were treated with diltiazem (50 µM) after the development of stable phasic contractions. Before diltiazem treatment, basal force was elevated in urothelium-intact strips relative to denuded strips (1.05 ± 0.04 mN, *n* = 6 vs. 0.81 ± 0.08 mN, *n* = 5, *P* < 0.05). Diltiazem was applied to assess the level of tone. Diltiazem relaxed urothelium-intact and denuded strips to the same level of force (0.83 ± 0.04 mN, *n* = 6 vs. 0.67 ± 0.10 mN, *n* = 5, *P* > 0.05). Since indomethacin and diltiazem both relax force by the same degree in urothelium-intact and denuded strips, it is possible that COX-derived prostanoids enhance bladder smooth muscle tone and phasic contractions by increasing Ca^2+^ entry through voltage-dependent Ca^2+^ channels.

### Contribution of Prostanoids to Transient Pressure Events in Isolated Pressurized Bladders

**Figure 7.**
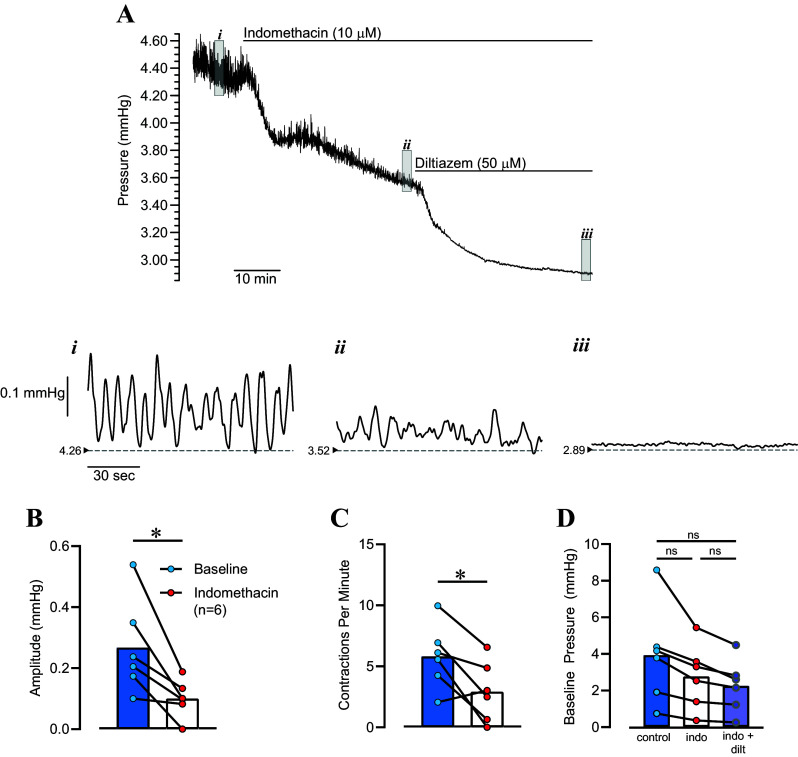
Inhibition of transient pressure events in isolated pressurized urinary bladders by indomethacin. *A*: Original recording of bladder pressure in a bladder maintained at a volume of 80% capacity before and after applying indomethacin and diltiazem. *Insets* show expanded view of bladder pressure under baseline conditions (*i*), after applying indomethacin (*ii*), and after applying diltiazem in continued presence of indomethacin (*iii*). The small arrowheads and dashed lines indicate basal pressure in each condition. Summary data show that the average contraction amplitude (*B*) and frequency (*C*) decreased in the presence of indomethacin. Baseline pressure under each condition is shown in *D*. Individual data points are shown with a line connecting paired observations. *P < 0.05 by paired *t* test; one-way repeated-measures ANOVA with Šidák’s test for multiple comparisons. ns, not significant.

In isolated pressurized bladders, the spontaneous transient contractions seen in bladder strips manifest as transient pressure events during bladder filling (24). We hypothesized that transient pressure events would be similarly inhibited by indomethacin. We filled isolated bladders to a volume of 80% capacity and allowed them to stabilize for 30–60 min under isovolumic conditions, at which point indomethacin (10 µM) was applied (Fig. 7). Transient pressure events occurred at a regular frequency under baseline conditions. Indomethacin decreased bladder pressure from 3.93 ± 1.10 mmHg to 2.77 ± 0.73 mmHg) (*n* = 6; *P* = 0.08 one-way repeated measures analysis of variance with Tukey’s test for multiple comparisons). In addition, transient pressure event amplitude decreased from 0.27 ± 0.06 mmHg to 0.10 ± 0.03 mmHg (*P* < 0.05), whereas frequency decreased from 5.8 ± 1.1 contractions per minute to 2.9 ± 1.0 contractions per minute (*P* < 0.05). The voltage-dependent Ca^2+^ channel (VDCC) blocker diltiazem (50 µM) further decreased bladder pressure to 2.26 ± 0.59 mmHg (*P* < 0.05 vs. indomethacin, *P* = 0.054 vs. baseline) and eliminated transient pressure events (Fig. 7).

### Effects of COX Inhibition on Bladder Afferent Nerve Activity during Filling

**Figure 8.**
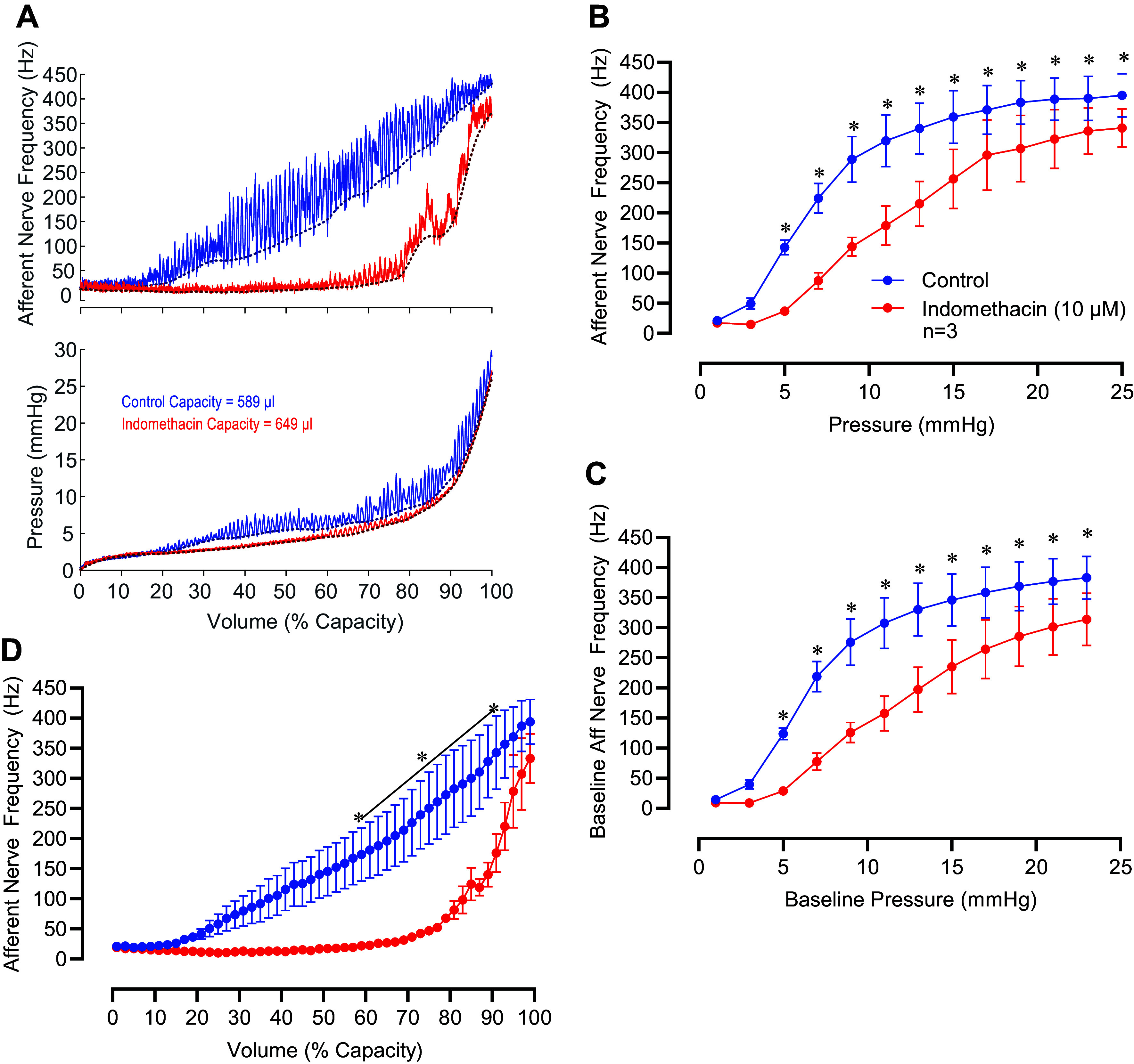
Indomethacin decreases afferent nerve discharge frequency in isolated pressurized urinary bladders during bladder filling. *A*: original recordings of afferent nerve discharge frequency (*top*) and pressure (*bottom*) in a mouse urinary bladder during filling from empty volume to 100% capacity before (blue trace) and after (red trace) applying indomethacin. The *x*-axes are normalized to 100% capacity, and the bladder volumes at 100% capacity during the control recording and indomethacin recording are indicated in the *bottom* panel. Dashed lines below the raw traces indicate baseline measurements obtained by fitting the nerve and pressure recordings using a weighted asymmetric least squares procedure (see methods). *B*: summary data showing the average afferent nerve discharge frequency measured over 2-mmHg increments as the bladder was filled up to 25 mmHg. *C*: baseline afferent nerve discharge frequency measured over 2-mmHg increments. *D*: average afferent nerve discharge frequency expressed as a function of bladder capacity. For *B* through *D*, results are means ± SE with *P< 0.05 by two-way repeated-measures analysis of variance followed by Šidák’s test for multiple comparisons.

**Figure 9.**
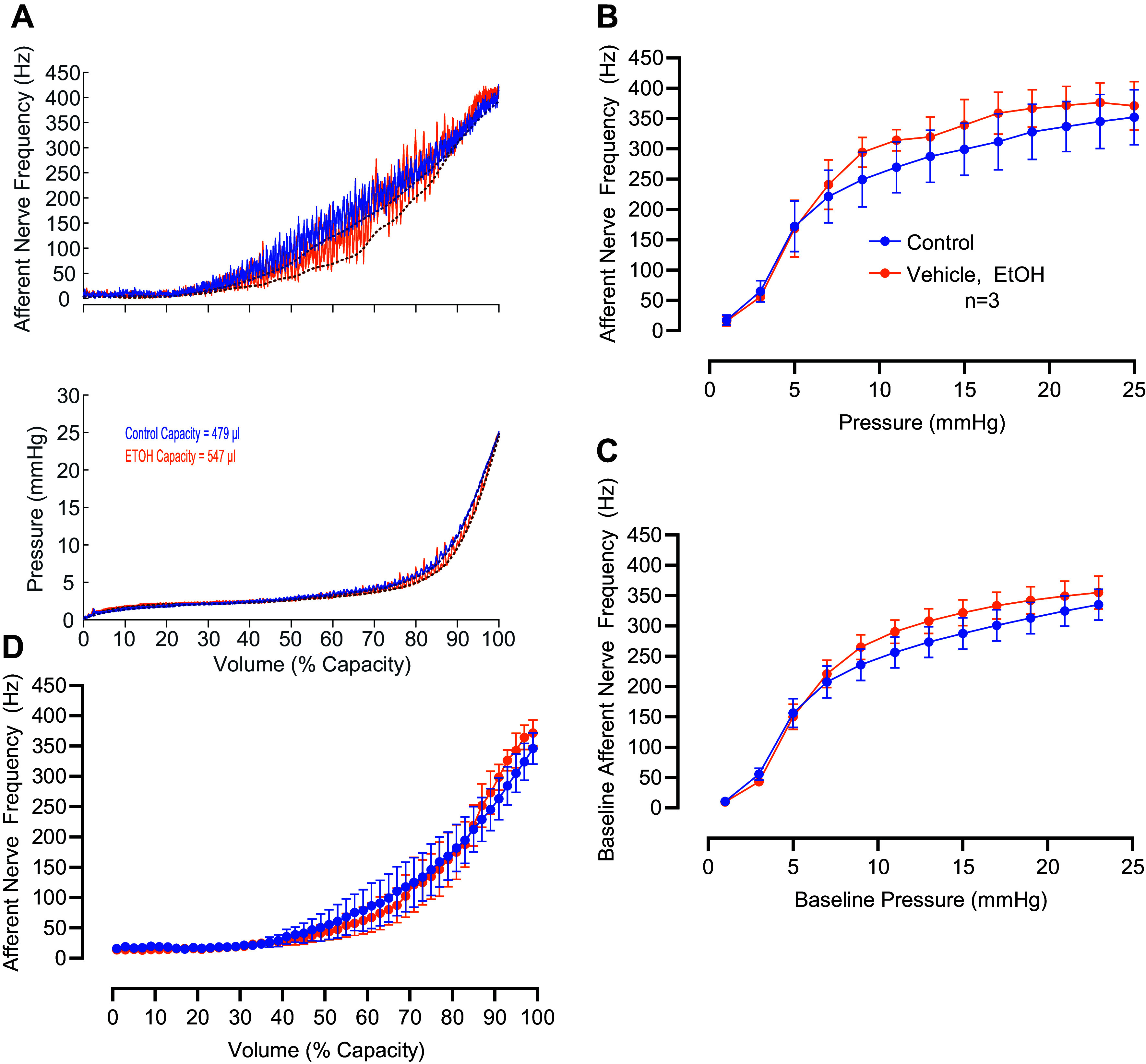
Afferent nerve discharge frequency in isolated pressurized urinary bladders during bladder filling is unaltered by the indomethacin vehicle ethanol (EtOH, 0.01%). *A*: original recordings of afferent nerve discharge frequency (*top*) and pressure (*bottom*) in a mouse urinary bladder during filling from empty volume to 100% capacity before (blue trace) and after (orange trace) applying the indomethacin vehicle. The *x*-axes are normalized to 100% capacity, and the bladder volumes at 100% capacity during the control recording and vehicle recording are indicated in the *lower* panel. Dashed lines below the raw traces indicate baseline measurements obtained by fitting the nerve and pressure recordings using a weighted asymmetric least squares procedure (see methods). *B*: summary data showing the average afferent nerve discharge frequency measured over 2-mmHg increments as the bladder was filled up to 25 mmHg. *C*: baseline afferent nerve discharge frequency measured over 2-mmHg increments. *D*: average afferent nerve discharge frequency expressed as a function of bladder capacity. For *B* through *D*, results are means ± SE, and there were no significant differences.

A separate series of experiments was performed in which we measured afferent nerve activity during filling cycles in the ex vivo pressurized bladder preparation. Under baseline conditions, bladder capacity was 0.545 ± 0.064 mL (*n* = 5). After treatment with indomethacin, bladder capacity increased significantly to 0.604 ± 0.069 mL (*n* = 5, *P* = 0.0350, two-tailed paired *t* test). In the ex vivo bladder preparation, we consistently observe a slight increase in bladder capacity over successive filling cycles (24, 39). To account for this phenomenon, a separate set of bladders was treated with the indomethacin vehicle, ethanol (0.1%). Before being treated with ethanol, bladder capacity was 0.502 ± 0.041 mL. After ethanol treatment, bladder capacity increased to 0.536 ± 0.050 mL (*n* = 3, *P* = 0.222, two-tailed paired *t* test). Since there is a gradual increase in bladder compliance over time, we expressed afferent nerve activity and bladder pressure as a function of normalized bladder volume (Figs. 8*A* and 9*A*).

During filling, bladder pressure remained relatively low, up to a point of about 80% capacity when pressure began to rise steeply (Figs. 8*A* and 9*A*, *bottom*). Small transient increases in bladder pressure (transient pressure events) occurred at regular intervals. Transient pressure events were associated with concomitant bursts of afferent nerve activity (Figs. 8*A* and 9*A*, *top*, also see Ref. 24). Afferent nerve activity was averaged within incremental 2 mmHg bins through the filling phase (Fig. 8*B*). Inhibition of prostaglandin synthesis with indomethacin decreased the appearance of transient pressure events (Fig. 8*A*) and greatly decreased the amount of afferent nerve activity observed (Fig. 8*B*). Since transient pressure events trigger bursts of afferent nerve activity, and indomethacin inhibits transient pressure events, it is possible that the indomethacin-induced decrease in afferent nerve activity simply reflects the decrease in the amplitude of transient pressure events. To address this possibility, we fitted the nerve and pressure recordings with a least squares baseline estimation model (30). When we examined the relationship between the baseline afferent nerve signal and the baseline pressure, we still observed a robust and significant decrease in nerve activity following indomethacin treatment (Fig. 8*C*). Afferent nerve activity and transient pressure events remained stable in control experiments where the vehicle for indomethacin (ethanol, 0.01%) was applied instead of indomethacin (Fig. 9).

## DISCUSSION

**Figure 10.**
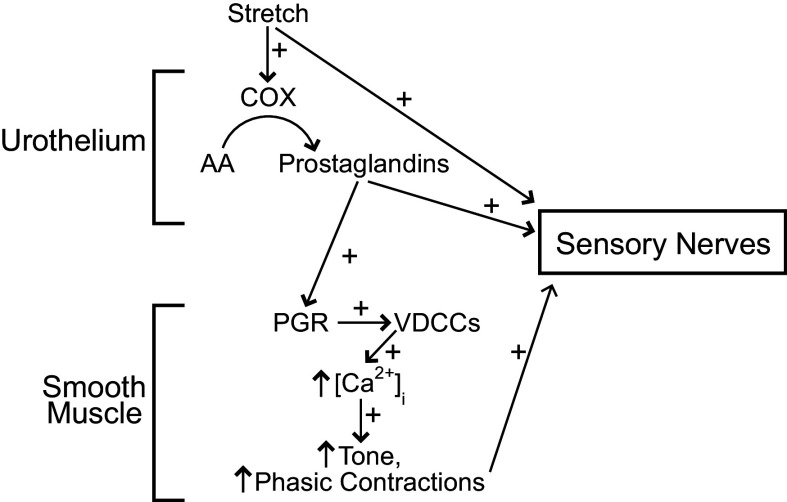
Proposed scheme outlining how urothelium-derived prostanoids increase bladder smooth muscle contractility. Mechanical stretch that occurs during bladder filling may trigger the release of cyclooxygenase (COX)-derived prostanoids/prostaglandins from the urothelium. COX produces prostaglandins from arachidonic acid (AA). Urothelial-derived prostaglandins activate prostaglandin receptors (PGR) in the underling bladder smooth muscle cells. Activation of PGRs triggers Ca^2+^ entry through voltage-dependent Ca^2+^ channels (VDCCs). The resulting phasic contractions of the detrusor in turn trigger bursts of afferent nerve activity due to mechanical wall deformation (e.g., Ref. 24). In addition, urothelial-derived prostaglandins may directly activate sensory nerves.

In the present study, we found that mouse urinary bladder strips develop more tone and larger, more frequent phasic contractions in the presence of urothelium. This urothelium-dependent increase in basal tone and phasic contractility was independent of nerves and could not be attributed to differences in the ability of the tissues to develop force. Blocking COX with indomethacin and inhibiting voltage-dependent Ca^2+^ channels with diltiazem completely abolished the increase in basal tone and phasic contractions seen in urothelium-intact bladder strips. Furthermore, blocking COX inhibited transient pressure events and greatly reduced afferent nerve output during bladder filling in isolated pressurized mouse urinary bladders. In the present study, we only examined bladders from male mice. However, we have examined transient pressure events in bladders from female mice as part of an unrelated study (unpublished), and they appear indistinguishable from those observed in male mouse bladders, so we do not anticipate any sex differences in the appearance of phasic contractions of bladder smooth muscle. Taken together, these results suggest that urothelium-derived PGs or other COX-derived prostanoids may play a role in contributing to detrusor contractile activity and sensory nerve output during normal voiding. Figure 10 summarizes our working hypothesis for how urothelial-derived prostanoids affect UBSM contractility and sensory nerve activity. We suggest that PGs may be released by the urothelium via the activity of COX, and that mechanical stretch that occurs as the bladder fills with urine may be a stimulus to enhance the release of PGs from the urothelium. PGs would then activate PG receptors on the underlying UBSM, where they would trigger phasic contractions attributed to the opening of VDCCs. In the whole bladder, these transient contractions would translate to transient pressure events, which trigger bursts of afferent nerve activity. In addition, our results suggest that PGs may have a direct excitatory effect on afferent nerves in the bladder.

Several studies have indicated that the urothelium is a source of substances that exert a relaxing influence on urinary bladder smooth muscle (11, 15–17, 20). In urothelium-intact pig urinary bladder strips, contractile responses to muscarinic agonists (11, 20) and histamine (20) are blunted. In addition, spontaneous phasic contractions are smaller in amplitude in urothelium-intact pig bladder strips compared with urothelium-denuded strips (40). Contractile responses to substance P are blunted in urothelium-intact guinea pig bladder strips (17). In the cat urinary bladder, removal of the urothelium potentiated the contractile responses to field stimulation, muscarinic agonists, ATP, and potassium chloride (16). Since the urothelium is a source of NO (41), NO would seem to be a likely candidate to relax urinary bladder smooth muscle, analogous to its role in the vascular endothelium. However, blocking NO synthesis fails to prevent the action of the putative urothelium-derived relaxing factor (11, 15). At present, there is no clear indication of what this/these factor/s may be. Furthermore, variations in study conditions and species studied may account for the apparent differences in urothelial function.

In contrast to a relaxing influence of the urothelium, other studies suggest that the urothelium is a source of substances that elicit UBSM contraction (14, 19, 42). Downie and Karmazyn (14) showed that rabbit bladder urothelium releases prostaglandins in response to mechanical perturbation. When samples of bathing media from urothelial strips that had been agitated with a rod were applied to denuded urinary bladder strips, basal tension and electric field stimulation-induced contractions were augmented (14). Furthermore, direct application of PGE1, PGE2, and PGF2α causes contraction of bladder smooth muscle that could be blocked by inhibiting voltage-dependent Ca^2+^ channels (33). Another report by Pinna and coworkers (18) found that bradykinin-induced contractions are augmented in diabetic rat bladders, and this effect could be partially attributed to urothelium-derived prostanoids that mediated UBSM contraction. Similar results were seen indicating a role for urothelium-derived contracting factors in modulating responses to substance P and capsaicin in bladders in diabetic rats (42).

Spontaneous phasic contractions observed in isolated strips of urinary bladder appear to underlie the transient pressure events observed in isolated pressurized bladder preparations, and these may underlie nonvoiding contractions observed with urodynamics clinically (24, 43, 44). Since PGs seem to modulate these contractions, it is possible that these contractions may contribute to bladder overactivity seen in pathological conditions where PG levels in the bladder are elevated. Indeed, PGs have been shown to be associated with bladder overactivity in a rat model of interstitial cystitis (45) and in human females with overactive bladder (46).

Indomethacin may block VDCCs (37). However, those studies showed that a relatively high concentration of indomethacin (300 µM), while completely blocking phasic contractions of human uterine smooth muscle, only blocked VDCC current by about 40%. Furthermore, the much lower concentration of indomethacin that we used in the present study (10 µM) only blocked VDCC current by 5–10%, and this did not affect uterine contractility (37).

Membrane depolarization may also increase the affinity of prostaglandin receptors for prostanoids (47). So as the UBSM membrane becomes more excitable during action potential bursting, any endogenous prostaglandins may have a more pronounced effect on augmenting phasic contractility.

The role of urothelial PGs in modulating bladder function is generally considered in the context of overactive bladder or bladder inflammation, where it is thought that dampening urothelial PG activity through COX-1 inhibition or PG receptor antagonists may be a treatment for overactive bladder. However, our results suggest that urothelial PGs may play a role in normal bladder physiology. Takagi-Matsumoto and colleagues, while examining the potential use of nonsteroidal anti-inflammatory drugs (NSAIDs) in treating bladder overactivity induced by cyclophosphamide cystitis, made some interesting observations regarding NSAIDs under normal conditions (48). They found in normal rats that intravenous administration of indomethacin or ketoprofen decreased the frequency of transient contractions during bladder filling. In addition, when conducting cystometry, they found that ketoprofen and indomethacin increased bladder capacity significantly (48), suggesting that blocking COX pathways may result in altered sensory function during filling under normal conditions. In addition, a clinical study conducted in the Netherlands showed that men taking nonsteroidal anti-inflammatory drugs had a twofold higher risk of manifesting urinary retention relative to patients not taking NSAIDs (49). This is consistent with our current observations where inhibiting COX with indomethacin attenuates transient phasic contractions and decreases afferent sensory nerve activity during bladder filling.

The results from our present study indicate that urothelium-intact bladder strips develop more basal tone and larger, more frequent phasic contractions than denuded strips following a stretch stimulus (Figs. 1 and 2). The increase in contractility observed in urothelium-intact bladder strips is abolished following inhibition of COX or blockade of voltage-dependent Ca^2+^ channels (Figs. 4, 5, 7, and 8). It is possible that bladder distension promotes the production of prostaglandins or other prostanoids by the urothelium. The urothelium-derived prostanoids activate receptors on urinary bladder smooth muscle cells, promoting Ca^2+^ entry through voltage-dependent Ca^2+^ channels, raising intracellular Ca^2+^ concentration, and causing an increase in tone and phasic contractions (Figs. 5 and 7). The indomethacin-sensitivity of urothelium-denuded bladder strips (Fig. 5) suggests that bladder smooth muscle cells, or other cell types in the bladder wall that remain following removal of the urothelium, are also sources of prostaglandins. Finally, urothelial-derived prostanoids also appear to activate sensory nerves in the bladder wall, as blocking COX greatly attenuates sensory nerve output during bladder filling (Fig. 8).

## DATA AVAILABILITY

Data will be made available upon reasonable request.

## GRANTS

This work was supported by National Institute of Diabetes and Digestive and Kidney Diseases Grant R01DK125543 (to T.J.H. and G.M.H.).

## DISCLOSURES

G.M.H. is a scientific consultant at MED Associates, Inc. and Living Systems Instrumentation, a division of Catamount Research and Development, Inc., and his wife is a co-owner of these companies. None of the other authors has any conflicts of interest, financial or otherwise, to disclose.

## AUTHOR CONTRIBUTIONS

G.M.H. conceived and designed research; T.J.H., H.J.F., J.L.R., E.M.B., and G.M.H. performed experiments; T.J.H., H.J.F., J.L.R., and G.M.H. analyzed data; T.J.H., G.W.H., M.T.N., and G.M.H. interpreted results of experiments; T.J.H., J.L.R., and G.M.H. prepared figures; G.M.H. drafted manuscript; T.J.H., J.L.R., G.W.H., M.T.N., and G.M.H. edited and revised manuscript; T.J.H., H.J.F., J.L.R., E.M.B., G.W.H., M.T.N., and G.M.H. approved final version of manuscript.
